# Supplementary material for: Electrochemical Behavior of Some Cinchona Alkaloids Using Screen-Printed Electrodes
Source: Sensors (Basel). 2025 Apr 1;25(7):2216. doi: 10.3390/s25072216 (PMC11991525; doi:10.3390/s25072216)
Supplement: Supplementary file 1 [file sensors-25-02216-s001.zip › sensors-3522612-supplementary.pdf]

Electronic Supplementary Information (ESI)

**Electrochemical Behavior of Some Cinchona Alkaloids Using Screen-Printed Electrodes**

Tonino Caruso [1,*] and Laura Palombi [2]

[1] Department of Chemistry and Biology, University of Salerno, 84084 Fisciano, Italy;
[2] Department of Physical and Chemical Sciences, University of L'Aquila, 67100 Coppito, Italy

*Corresponding email: tcaruso@unisa.it

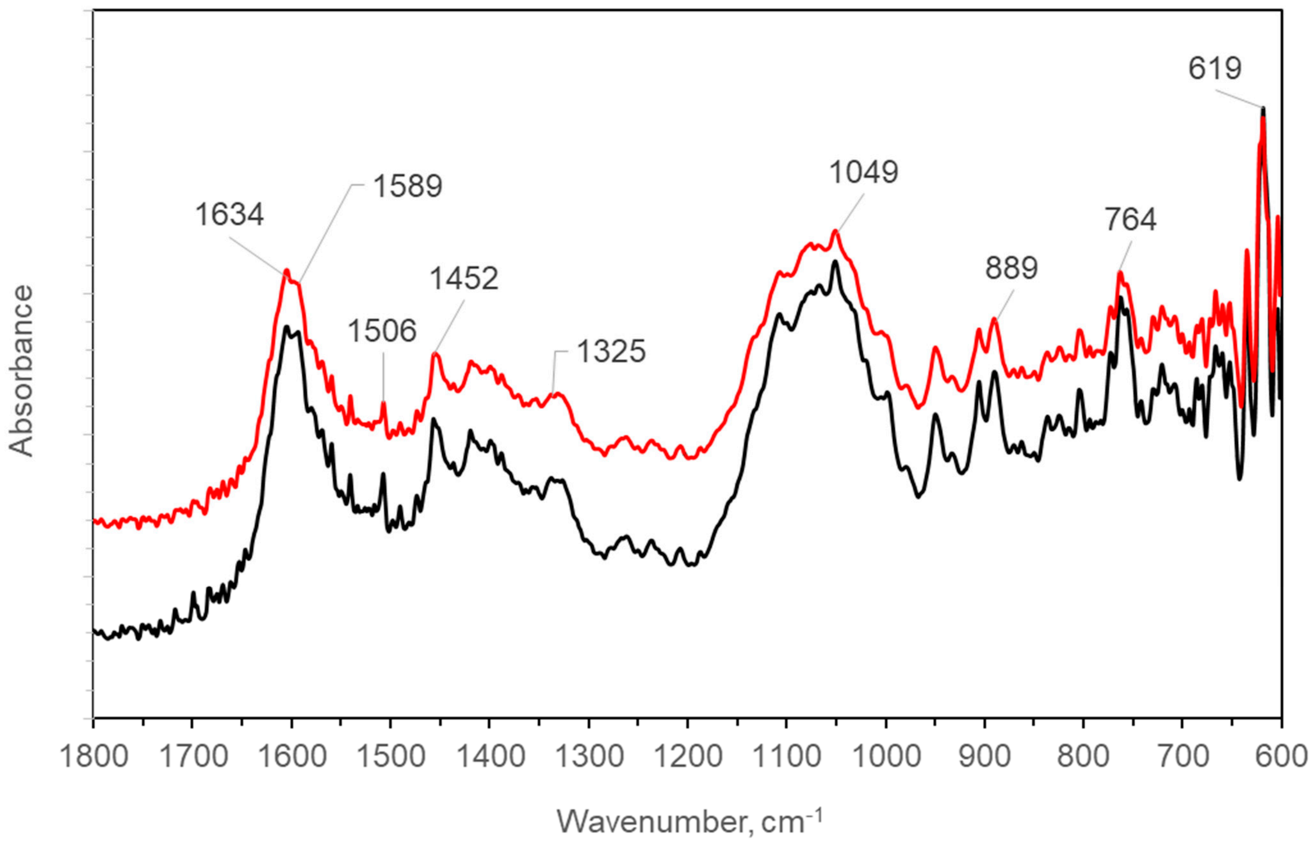


**Figure S1**: ATR-FTIR spectra of pure cinchonine (red) and deposited layers on Pt surfaces (black), dissolved in the same $CCl_4$ solution and concentrated drop by drop onto a ZnSe crystal, placed in the sample compartment of an ATR sampling module of the Cary 630 FTIR spectrophotometer. The spectra match. Vibrational assignments of the infrared spectra of pure cinchonine and cinchonine desorbed from platinum surfaces, reported in $cm^{-1}$, match those reported by Zaera.[1]

Acquisition Parameters: Sample Scans: 128. Background Scans: 8. Resolution: 4 $cm^{-1}$. Spectral Range: 1800-600 $cm^{-1}$. Sampling Technology: ATR. Sampling Subtype: ZnSe 1-Bounce. Detector Type: DTGS.

---

[1] Ma, Z.; Lee, I.; Zaera, F. Factors Controlling Adsorption Equilibria from Solution onto Solid Surfaces: The Uptake of Cin-chona Alkaloids on Platinum Surfaces. *J. Am. Chem. Soc.* **2007**, 129, 51, 16083–16090. https://doi.org/10.1021/ja076011a

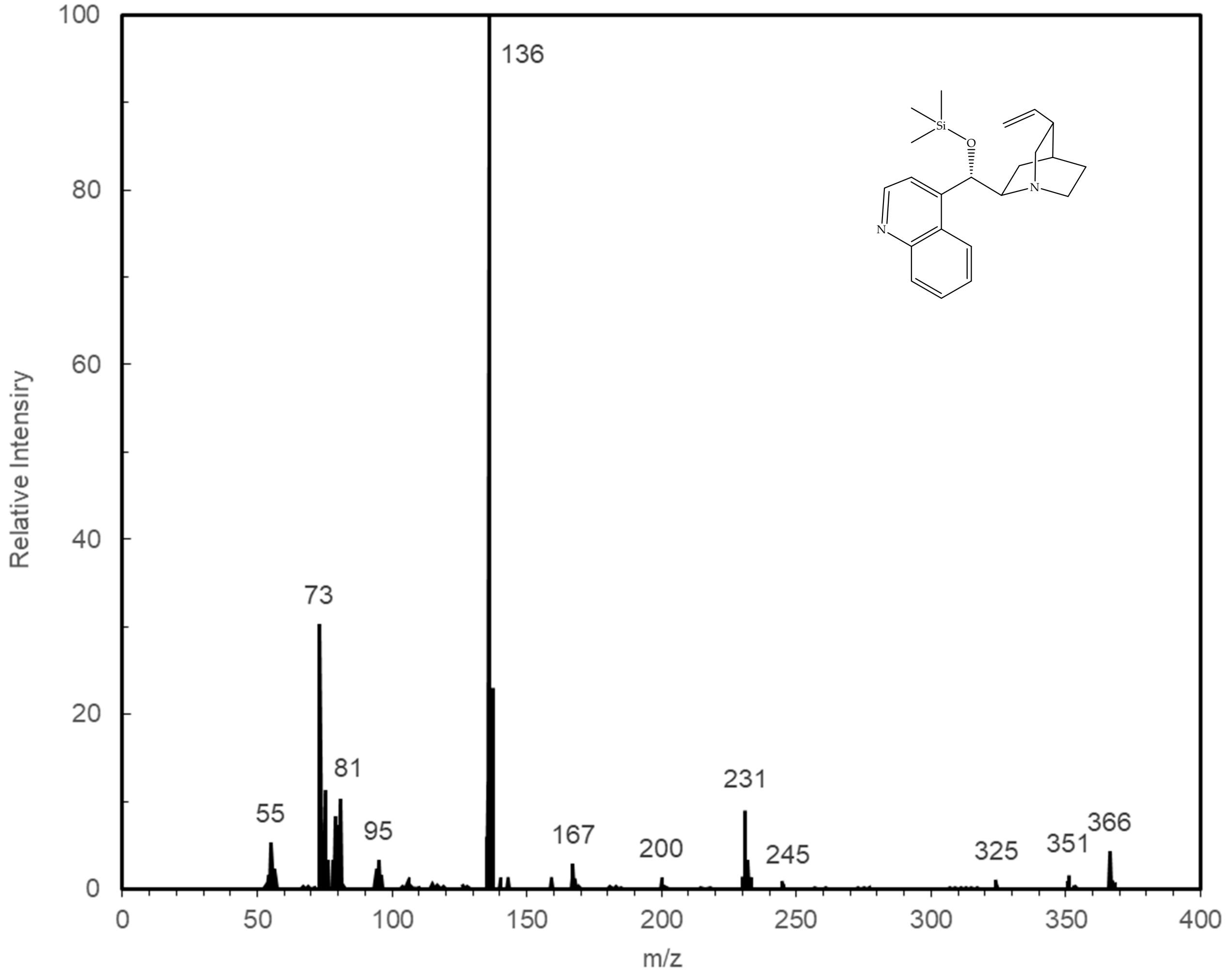


**Figure S2:** Mass spectrum (EI, 70 eV) of the deposited layers on Pt surfaces, dissolved in a $CCl_4$ solution, derivatized with BSTFA/$Et_3N$, and injected into a GC-MS equipped with an HP-5MS column (30 m × 0.25 mm ID × 0.25 μm film thickness). The MS spectrum corresponds to that of 9-O-(trimethylsilyl)cinchonine, as identified in the NIST Mass Spectral Library and also reported in the literature.[2]

[2] Isidorov, V.A. GC-MS of Biologically and Environmentally Significant Organic Compounds; TMS Derivatives; MS spectrum of Cinchonine, monoTMS, pag. 481. John Wiley & Sons: Hoboken, NJ, USA, 2020.

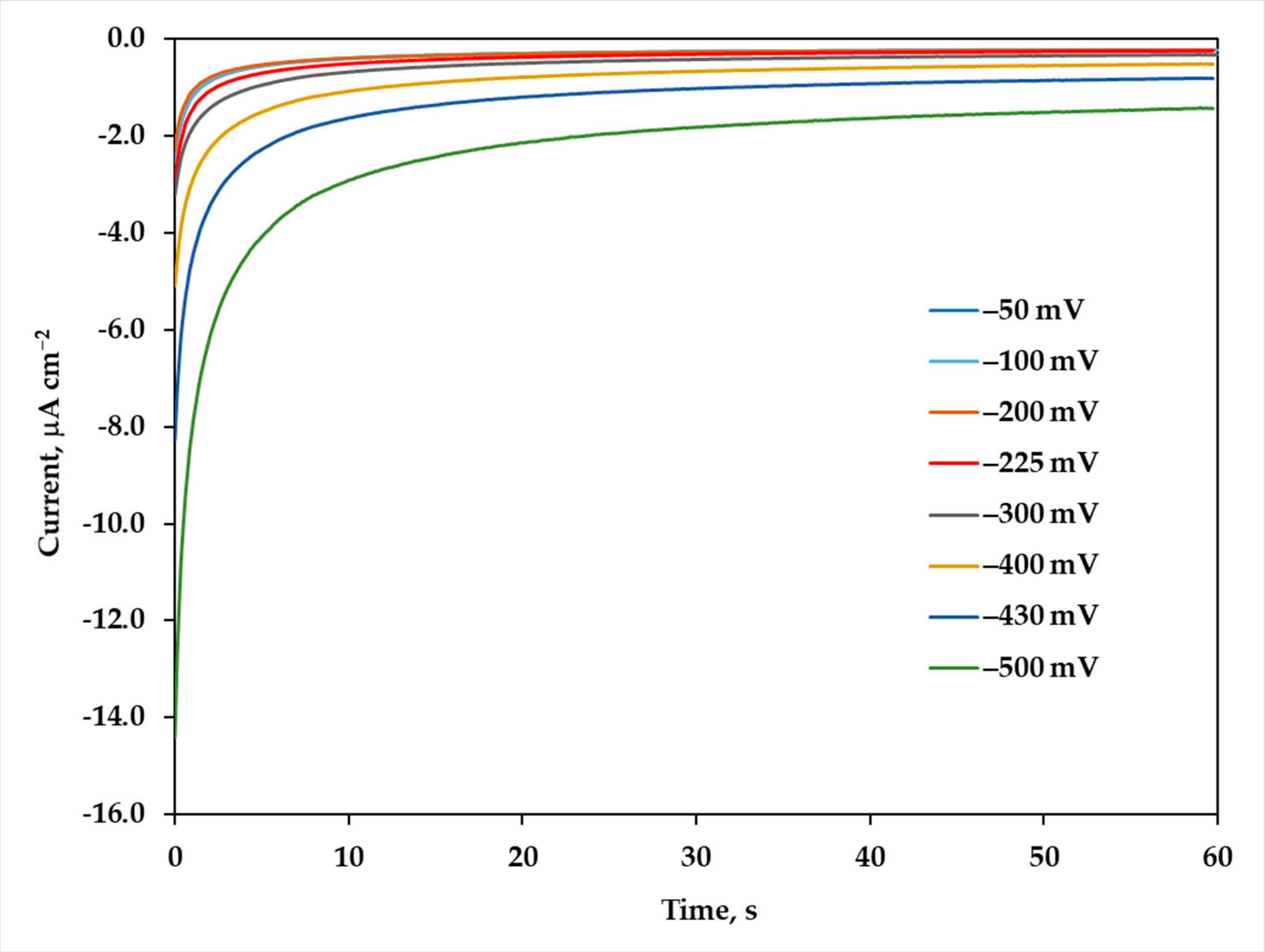


**Figure S3**: Chronoamperometric curves recorded at deposition potentials of -50 mV, -100 mV, -200 mV, -225 mV, -300 mV, -400 mV, -430 mV, and -500 mV.
